# Information-theoretic equilibrium and observable thermalization

**DOI:** 10.1038/srep44066

**Published:** 2017-03-07

**Authors:** F. Anzà, V. Vedral

**Affiliations:** 1 Atomic and Laser Physics, Clarendon Laboratory, University of Oxford, Parks Road, Oxford, OX1 3PU, UK; 2 Centre for Quantum Technologies, National University of Singapore, 117543, Singapore; 3 Department of Physics, National University of Singapore, 2 Science Drive 3, 117551, Singapore; 4 Center for Quantum Information, Institute for Interdisciplinary Information Sciences, Tsinghua University, 100084, Beijing, China

## Abstract

A crucial point in statistical mechanics is the definition of the notion of thermal equilibrium, which can be given as the state that maximises the von Neumann entropy, under the validity of some constraints. Arguing that such a notion can never be experimentally probed, in this paper we propose a new notion of thermal equilibrium, focused on observables rather than on the full state of the quantum system. We characterise such notion of thermal equilibrium for an arbitrary observable via the maximisation of its Shannon entropy and we bring to light the thermal properties that it heralds. The relation with Gibbs ensembles is studied and understood. We apply such a notion of equilibrium to a closed quantum system and show that there is always a class of observables which exhibits thermal equilibrium properties and we give a recipe to explicitly construct them. Eventually, an intimate connection with the Eigenstate Thermalisation Hypothesis is brought to light.

To understand under which conditions thermodynamics emerges from the microscopic dynamics is the ultimate goal of statistical mechanics. However, despite the fact that the theory is more than 100 years old, we are still discussing its foundations and its regime of applicability. The ordinary way in which thermal equilibrium properties are obtained, in statistical mechanics, is through a complete characterisation of the thermal form of the state of the system. One way of deriving such form is by using *Jaynes principle*^1^,^2^,^3^,^4^, which is the constrained maximisation of von Neumann entropy *S*_vN_ = −Tr*ρ* log*ρ*. Jaynes showed that the unique state that maximises *S*_vN_ (compatibly with the prior information that we have on the system) is our best guess about the state of the system at the equilibrium. The outcomes of such procedure are the so-called *Gibbs ensembles*.

In the following we argue that such a notion of thermal equilibrium, *de facto* is not experimentally testable because it gives predictions about all possible observables of the system, even the ones which we are not able to measure. To overcome this issue, we propose a weaker notion of thermal equilibrium, specific for a given observable.

The issue is particularly relevant for the so-called “Pure states statistical mechanics”^5^,^6^,^7^,^8^,^9^,^10^,^11^,^12^,^13^,^14^,^15^,^16^,^17^,^18^,^19^, which aims to understand how and in which sense thermal equilibrium properties emerge in a closed quantum system, under the assumption that the dynamic is unitary. In the last fifteen years we witnessed a revival of interest in these questions, mainly due to remarkable progresses in the experimental investigation of isolated quantum systems^20^,^21^,^22^,^23^,^24^,^25^. The high degree of manipulability and isolation from the environment that we are able to reach nowadays makes possible to experimentally investigate such questions and to probe the theoretical predictions.

The starting point of Jaynes’ derivation of statistical mechanics is that *S*_vN_ is a way of estimating the uncertainty that we have about which pure state the system inhabits. Unfortunately we know from quantum information theory that it does not address all kind of ignorance we have about the system. Indeed, it is not the entropy of an observable (though the state is observable); its conceptual meaning is not tied to something that we can measure.

This issue is intimately related with the way we acquire information about a system, i.e. via measurements. The process of measuring an observable 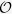 on a quantum system allows to probe only the diagonal part of the density matrix 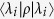, when this is written in the observable eigenbasis 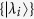. For such a reason, from the experimental point of view, it is not possible to assess whether a many-body quantum system is at thermal equilibrium (e.g. Gibbs state *ρ*_*G*_): the number of observables needed to probe all the density matrix elements is too big. In any experimentally reasonable situation we have access only to a few (sometimes just one or two) observables. It is therefore natural to imagine situations in which the outcomes of measurements are compatible with the assumption of thermal equilibrium, while the rest of the density matrix of the system is not.

Despite that, we think that the fact that a distribution is compatible with its thermal counterpart will lead to the emergence of certain thermal properties, concerning the specific observable under scrutiny. Building our intuition on that, we propose a new notion of thermal equilibrium specific for a given observable, experimentally verifiable and which relies on a figure of merit that is not the von Neumann entropy. A good choice for such a figure of merit comes from quantum information theory and it is the Shannon entropy 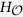 of the eigenvalues probability distribution {*p(λ*_*j*_)} of an observable 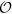. The well-known operational interpretation of 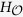
26 matches our needs since it addresses the issue of the knowledge of an observable and it provides a measure for the entropy of its probability distribution.

Throughout the paper we will work under the assumption that the Hilbert space of the system has finite dimension and we will refer to the case in which the Hamiltonian of the system has no local conserved quantities, even though it is possible to address situations where there are several conserved quantities, like integrable quantum systems. We will also assume that the observable has a pure-point spectrum with the following spectral decomposition 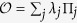, where Π_*j*_ is the projector onto the eigenspace defined by the eigenvalue *λ*_*j*_. 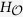 is the entropy of its eigenvalues probability distribution *p(λ*_*j*_) ≡ Tr(*ρ*Π_*j*_)


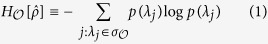


where 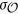 is the spectrum of 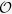.

We propose to define the notion of thermal equilibrium, for an arbitrary but fixed observable 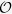, via a characterisation of the probability distribution of its eigenvalues. We will say that


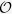
 *is at thermal equilibrium when its eigenvalues probability distribution p(λ*_*j*_) *maximises the Shannon entropy* 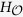, *under arbitrary perturbations with conserved energy*. We call an observable with such a probability distribution: *thermal observable*.

It is important to note that this notion characterises only the probability distribution at equilibrium and it does not uniquely identify an equilibrium state. Given the equilibrium distribution *p(λ*_*j*_) = *p*^eq^(*λ*_*j*_), there will be several quantum states *ρ* which give the same probability distribution for the eigenvalues *λ*_*j*_. In this sense this is a weaker notion of equilibrium, with respect to the ordinary one.

In the rest of the paper we study the main consequences of the proposed notion of observable-thermal-equilbrium: its physical meaning and the relation with Gibbs ensembles. The investigation will show that the proposed notion of equilibrium is able to address the emergence of thermalisation. This is our first result.

Furthermore, we study the proposed notion of equilibrium in a closed quantum system and prove that there is a large class of bases of the Hilbert space which always exhibit thermal behaviour and we give an algorithm to explicitly construct them. We dub them *Hamiltonian Unbiased Bases* (HUBs) and, accordingly, we call an observable which is diagonal in one of these bases *Hamiltonian Unbiased Observable* (HUO). The existence and precise characterisation of observables which always thermalise in a closed quantum system is our second result. Furthermore, we investigate the relation between the notion of thermal observable and one of the main paradigms of pure states statistical mechanics: the Eigenstate Thermalisation Hypothesis (ETH)^27^,^28^,^29^,^30^,^31^,^32^,^33^,^34^,^35^,^36^,^37^. We find an intimate connection between the concept of HUOs and ETH: the reason why these observables thermalise is precisely because they satisfy the ETH. Hence, with the existence and characterisation of the HUOs we are providing a genuine new prediction about which observables satisfies ETH, for any given Hamiltonian. The existence of this relation between HUOs and ETH is a highly non trivial feature and the fact that we can use it to predict which observables will satisfy ETH is our third result.

In the conclusive section we summarise the results and discuss their relevance for some open questions.

## Results

### Information-theoretic equilibrium

The request that the equilibrium distribution must be a maximum for 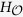 is phrased as a constrained optimisation problem and it is solved using the Lagrange multipliers technique. The details are given in in the Methods section. Two sets of equilibrium equations are obtained and we now show how they account for the emergence of thermodynamic behaviour in the observable 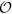.

We assume that the only knowledge that we have on the system is the normalisation of the state and the mean value of the energy 〈*T*〉 = *E*_0_, where *T* is the Hamiltonian of the system. The Hamiltonian has the following spectral decomposition *T* = ∑_*α*_*E*_*α*_*T*_*α*_, where 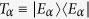 and we assume that its eigenvectors 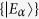 provide a full basis of the Hilbert space. We call 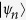 the eigenstates of the density operator, *ρ*_*n*_ are the respective projectors and *q*_*n*_ its eigenvalues. To describe the state of the system we use the following convenient basis: 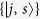 in which the first index *j* runs over different eigenvalues *λ*_*j*_ of 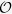 and the second index *s* accounts for the fact that there might be degeneracies. We also make use of the projectors 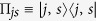. Furthermore, we define the following notation 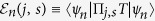. Using the overlaps 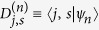 the constraints are:


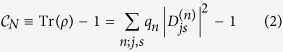



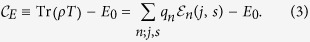


It is worth to note that the knowledge of *E*_0_ is always subject to uncertainty, which we call *δE*. In this sense, all the states 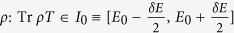 will be considered solutions of the constraint equation. Even though we do not make any assumption on *δE*, we note that it is usually assumed to be small on a macroscopic scale but still big enough to host a large number of eigenvalues of the Hamiltonian.

Exploiting Lagrange’s multipliers technique we obtain four sets of equations. Derivatives with respect to the multipliers enforce the validity of the constraints while the derivatives with respect to the overlaps give two independent set of equations. Using two linear combinations of them we obtain the following equilibrium equations (EEs):


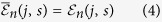



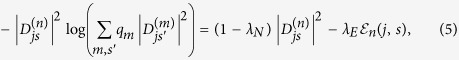


where the overbar indicates complex conjugation. *λ*_*E*_ and *λ*_*N*_ are the Lagrange multipliers associated to 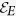 and 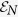, respectively.

The first step is to understand the physical meaning of these equations. The first one (Eq. (4)) gives the stability under the flow generated by the Hamiltonian and it implies that the equilibrium distribution *p*_eq_(*λ*_*j*_) has to be be invariant under the unitary dynamics. Indeed, writing the time evolution equation for *p(λ*_*j*_) we obtain


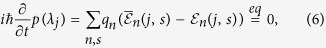


where the superscript “*eq*” stands for “after plugging in the EEs”.

The second equation (Eq. (5)) fixes the functional form of the distribution with respect to the Hamiltonian and to the Lagrange multipliers. It can be shown that it is responsible for the emergence of a thermodynamical relation between the entropy 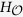 and the mean value of the energy. Integrating Eq. (5) over the whole spectrum of 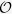 we obtain


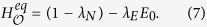


There is a linear contribution in the mean value of the energy, plus a “zero-point” term 
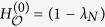
. This relation brings to light the thermodynamical relevance of Shannon entropy 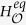 since such linear dependence on the average energy is a distinguishing feature of thermodynamic equilibrium. We note a strong analogy with the properties of von Neumann entropy, which acquires thermodynamical relevance once the state of the system is the Gibbs state 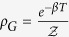, where 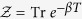 is the partition function


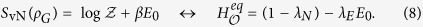


### Relation with statistical mechanics

We now come to the issue of understanding if our proposal is compatible with Gibbs ensembles. First we note that the ordinary notion of thermal equilibrium is much more stringent than ours, being a complete characterisation of the state of the system. Thus, we need to find the condition under which our criterion gives the full state of the system. Since we are using a maximum-entropy principle, a plausible auxiliary condition is the maximisation of the smallest among all Shannon entropies. Such a request fully characterises the state of the system because the lowest Shannon entropy of the state is unique: it is the one in which the density matrix is diagonal. Indeed, using the Schur-concavity of the Shannon entropy and the Schur-Horn theorem^26^ it is easy to prove that


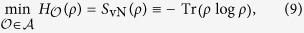


where 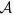 is the algebra of the observables^38^. Our minimalist request to maximise the lowest Shannon entropy is translated in the maximisation of von Neumann entropy, which gives Gibbs ensembles. It is therefore clear that our proposal constitutes an observable-wise generalisation of the ordinary notion of thermal equilibrium.

In the next section we will apply the proposed notion of thermal observable to the so-called “pure-states statistical mechanics”^5^,^6^,^7^,^8^,^9^,^10^ and we will investigate the relation with one of its main paradigm: the ETH.

### Closed Quantum Systems - Relation to ETH

ETH, in its original formulation^27^,^28^,^29^,^30^,^31^,^32^,^33^, is an ansatz on the matrix elements of an observable when it is written in the Hamiltonian eigenbasis 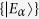:


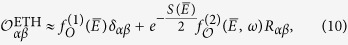


where 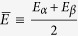, *ω* ≡ *E*_*α*_ − *E*_*β*_ while 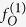 and 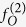 are smooth functions of their arguments. 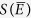 is the thermodynamic entropy at energy 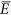 defined as 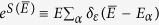, where *δ*_*ε*_ is a smeared version of the Dirac delta distribution. *R*_*αβ*_ is a complex random variable with zero mean and unit variance. Furthermore, we remember that ETH by itself does not guarantee thermalisation, we need to impose that the initial state has a small dispersion in the energy eigenbasis^30^. When this is true one says that 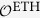 thermalises in the sense that its dynamically evolving expectation value is close to the microcanonical expectation value


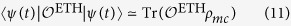


where *ρ*_*mc*_ = *ρ*_*mc*_(*E*_0_, *δE*) is the microcanonical state defined by the condition on the average value of the energy Tr*ρT* ∈ *I*_0_.

From the conceptual point of view, the “small energy-dispersion assumption” is a key element of the emergence of thermal equilibrium but it has nothing to do with ETH which, by itself, is only the ansatz in Eq. (10). Nevertheless, this assumption is expected to hold in real experiments because, when working with a many-body quantum system, it is almost impossible to prepare coherent superpositions of states with macroscopically different energies^18^,^19^.

Before we continue we define the following short-hand notation: 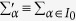, where *α* ∈ *I*_0_ means *α: E*_*α*_ ∈ *I*_0_.

#### Hamiltonian Unbiased Observables and ETH

To study the relation with ETH we need to change perspective. The point of view that we are adopting is the following. One of the key-points behind the ETH is that, in many real cases, the expectation values computed onto the Hamiltonian eigenvectors can be very close to the thermal expectation values. Moreover, when one wants to argue that thermalisation in a closed quantum system arises because of ETH, a main assumption is that the initial pure state of the system 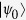 has a very small energy uncertainty Δ*E*, with respect to the average energy *E*_0_: 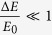. Following these two insights, we take the extreme limit in which Δ*E* = 0. With such a choice, we are left with an Hamiltonian eigenstate and a constraint equation given by Tr*ρH* = *E*_*α*_. We note that Eq. (4) is trivially satisfied for an Hamiltonian eigenstate. Hence we assume that 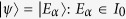 and use the solvability of Eq. (5) as a criterion to look for observables which can be thermal. While this is a very specific choice, we will show that it unravels some interesting features regarding the ETH.

With this assumption, the second equilibrium equation becomes


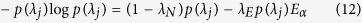


After crossing *p(λ*_*j*_) on both sides, the right-hand side does not depend on the label *j*. Hence, keeping in mind that *x*log*x* → 0 for *x* → 0, the most general solution of this equation is a constant distribution with support on some subset (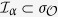 depending on *E*_*α*_) of the spectrum and zero on its complementary:


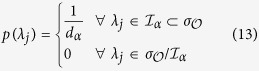


where 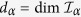 is the number of orthogonal states on which the distribution has non-zero value. The distribution of eigenvalues of 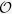 given by Eq. (13) fully agrees with the prediction from the microcanonical ensemble 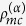, defined by the condition in Eq. (13). This is true, in particular, for the expectation value


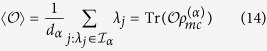


It has to be understood here that Eq. (13) is a highly non-trivial condition on 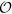, which is not going to be fulfilled by every observable since it imposes a very specific relation between its eigenstates and the energy eigenvectors.

A similar relation has already been studied within the context of quantum information theory. Two bases of a 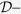
dimensional Hilbert space (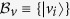 and 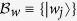) are called *mutually unbiased bases* (MUB)^39^,^40^,^41^ when


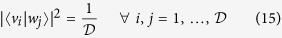


Such a concept is a generalisation, expressed in term of vector bases, of canonically conjugated operators. In other words, each vector of 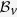 is completely delocalised in the basis 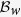 and viceversa. Here we mention the result about MUBs which matter most for our purposes: given the 2^*N*^-dimensional Hilbert space of *N* qubits, there are 2^*N*^ + 1 MUBs and we have an algorithm to explicitly find all of them^42^. Therefore, if we are in an energy eigenstate, an observable unbiased with respect to the Hamiltonian basis will always have a microcanonical distribution. This is also true if our state is not exactly an energy eigenstate, it is enough to have a state that has a sufficiently narrowed energy distribution. We now provide a simple argument to prove such a statement. We also note that such condition is closely related to the small dispersion condition briefly discussed before and that it is necessary to guarantee thermalisation, according to the ETH.

By assumption, the pure state in Eq. (??) has an energy distribution 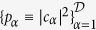 with small dispersion. This implies that its Shannon entropy *H*_*T*_ has a small value, because the profile of the distribution will be peaked around a certain value. For such a reason 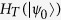 will be much smaller than its maximum value


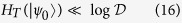


Moreover, it can be proven that between any pair of MUB, like the Hamiltonian eigenbasis and an HUB, there exists the following entropic uncertainty relation, involving their Shannon entropies^39^:


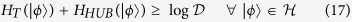


Putting together Eqs (16) and (17) we obtain that, for all the states with a small energy dispersion, the Shannon entropy of an HUB will always have a value close to the maximum:


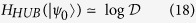


This in turn implies that the distribution of all the HUO will be approximately the same as the one computed on the microcanonical state.

For such a reason, we now study the properties of a HUO:


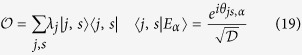


#### HUOs and ETH: diagonal matrix elements

In order to investigate the relation with ETH we need to study the matrix elements of a HUO in the energy basis:


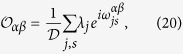


with 
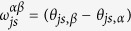
. It is straightforward to conclude that its diagonal matrix elements are constant in such a basis and therefore the so-called (Hamiltonian) Eigenstate Expectation values reproduce the microcanonical expectation values:


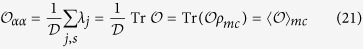


This is the first part of our third result and as it is, it can already be used to explain the emergence of thermalisation in a closed quantum system. In refs 18,19 Reimann proved an important theorem about equilibration of closed quantum systems. He was able to show that under certain conditions, the mean value of an observable is not much different from its value computed on the time-averaged density matrix, or Diagonal Ensemble (DE):


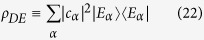


where *c*_*α*_ ≡ 〈*ψ*_0_|*E*_*α*_〉 and 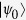 is the initial pure state of the isolated system. Roughly speaking, the two main assumptions made by Reimann are the following: first, that in any experimentally realistic condition the state of the system will occupy a huge number of energy eigenstates, even if the average energy is known up to a macroscopically small uncertainty; second, that the observable under study has a finite range of average values, due to the fact that we wish to measure it. For a clear and synthetic discussion on this topic we suggest^14^ and we send the reader to the original refs 18 and 19. We note that the first assumption does not contradict the small energy dispersion assumption. Indeed, as argued by Reimann, in a many-body quantum system, even if the energy is known up to a macroscopically small scale *δE*, there will be a huge number of eigenstates within the range *E*_*α*_ ∈ *I*_0_. To conclude, given a HUB it is always possible to obtain a HUO which satisfies the finite-range assumption. We can therefore apply Reimann’s theorem to HUOs.

It is important to note that Reimann’s theorem explains equilibration around the DE but this does not necessarily entail thermalisation. The DE still retains information about the initial state while thermalisation is defined (also) by the independence on the initial state. This is the point where our result is able to take a step forward and explain the emergence of thermal equilibrium in the HUOs. We can use Eq. (21) to prove that all the HUOs exhibit complete independence from the initial conditions:


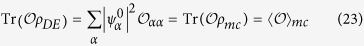


#### HUOs and ETH: off-diagonal matrix elements

In order to prove that a HUO satisfies ETH we need to study also its off-diagonal matrix elements. By using Eq. (19), we can investigate how the phases 
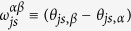
 are distributed. This can be done numerically, exploiting the available algorithms to generate MUBs^42^. The numerical investigation of the distribution of 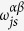 is reported in the Supplementary Material. Here we simply state the result: for a fixed value of the energy quantum numbers, the observed distributions of 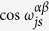, 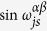 are well described by the assumption that 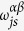 are independent and randomly distributed in [−*π, π*], with a constant probability distribution.

There are different ways in which this result can be used. The general argument is the following: in Eq. (20), the phases 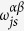 will have a randomising action on the eigenvalues *λ*_*j*_ and this will make the value of the off-diagonal matrix elements severely smaller than the value of the diagonal ones:


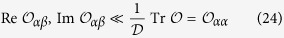


The randomness of the coefficients involved in the evaluation of the off-diagonal matrix elements has been recently proposed as the basic mechanism to explain the 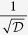 scaling behaviour which was observed to occur in some models^43^,^44^,^45^,^46^,^47^. The maximisation of Shannon entropy is therefore giving us a recipe to find the observables for which this is true.

#### HUOs and ETH: two important examples

In order to understand how this works in practice we need to say something specific about the eigenvalues. We highlight two important cases in which our result is helpful: an observable which is highly degenerate and an observable whose eigenvalues distribution is not correlated with the phases 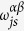.

##### Highly degenerate observable

Assuming that 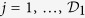 while 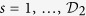 with 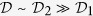, the sum in Eq. (20) splits into 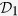 terms and each one of them is a sum of 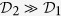 identically distributed random variables. We can apply the central limit theorem to the real and imaginary part of Eq. (20) and obtain the following expression for the off-diagonal matrix elements


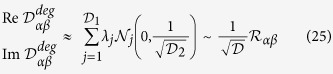


In which 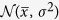 indicates a gaussian probability distribution with mean 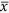 and variance *σ*^2^ and 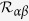 is a zero mean and unit variance random variable. This proves that a highly degenerate observable satisfies the ETH ansatz, Eq. (10). The diagonal matrix elements reproduce the microcanonical expectation values and the off-diagonal matrix elements are well described by a random variable with zero mean and 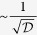 variance. This is precisely what we expect from a local observable, since its eigenvalues have a huge number of degeneracies, which grows exponentially with the size of the system, and it is in full agreement with the randomness conjecture made in ref. 46,47.

##### Uncorrelated distribution

If the eigenvalues distributions {*λ*_*j*_}_*j,s*_ and the phases 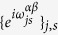 are not correlated Eq. (20) becomes


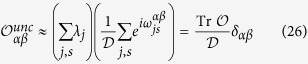


Where we used the fact that the two sequences are uncorrelated to approximate the sum as the product of two sums and in the second identity we used the fact that 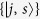 is a complete basis. We conclude that the off-diagonal matrix elements of such an observable are much smaller than the diagonal ones and therefore we can neglect them when we compute its dynamical expectation value:


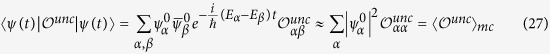


These results follows from the study of the equilibrium equations, under the assumption that the state is an Hamiltonian eigenstate 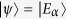 belonging to the energy shell *I*_0_. It is straightforward to see that the same results hold when the state is the microcanonical state *ρ*_*mc*_(*E*_0_,*δE*) involved in Eq. (11) and defined by the condition Tr*ρT* ∈ *I*_0_.

## Discussion

We proposed a new notion of thermal equilibrium for an observable 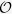: we say that 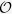 is a *thermal observable* when its eigenvalues probability distribution maximises its Shannon entropy 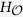. Setting up a constrained optimisation problem we derived two equilibrium equations and studied their physical implications. Eq. (4) enforces the stability of the distribution with respect to the dynamics generated by the Hamiltonian while Eq. (5) fixes the functional form of the distribution. Integrating the second equation we obtained a linear relation between Shannon entropy and the mean value of the energy, which shows that 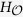 at equilibrium has thermodynamic properties. We also studied the relation of the proposed notion of equilibrium with quantum statistical mechanics. The request to maximise the lowest among all the possible Shannon entropies lead to Gibbs ensembles and therefore to the ordinary characterisation of thermal equilibrium. Together, the physical meaning of the equilibrium equations and the proven relation between maximisation of Shannon entropy and Gibbs ensemble, show that the maximisation of Shannon entropy is an observable-wise generalisation of the ordinary notion of thermal equilibrium. This is our first result.

In the second part of the paper we studied the emergence of thermal observables in a closed quantum system and especially their relation with the ETH. Using maximisation of 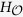 we were able to find a large class of observables which always thermalise and provide an algorithm to explicitly construct them. We call them Hamiltonian Unbiased Observables (HUOs). The existence and precise characterisation of a set of observables which always thermalises is our second result.

Using this characterisation, we studied the matrix elements of an HUO in the Hamiltonian eigenbasis. The study of the diagonal matrix elements reveals that they always satisfy ETH. This has been used to prove thermalisation of the average value of HUOs, in connection with Reimann’s theorem about equilibration of observables. The study of the off-diagonal matrix elements revealed that their value is exponentially suppressed, in the dimension of the Hilbert space. This completes the proof that HUOs satisfy the ETH. The proven relation between ETH and HUOs is our third and last result. The relevance of this result for the pure-states statical mechanics is related to the two main objections usually raised against ETH: the lack of predictive power for what concern both which observables satisfy ETH and how long it should take them to reach thermal equilibrium. The proposed notion of thermal equilibrium is therefore revealing its predictive power since it gives us a way of finding observables which always satisfy ETH, in a closed quantum system.

We would like to conclude by putting this set of results in a more general perspective. ETH is one of the main paradigms to justify the applicability of statistical mechanics to closed many-body quantum systems. However, it is just a working hypothesis, it is not derived from a conceptually clear theoretical framework. For such a reason, one of the major open issues is its lack of predictability. Despite that, there has been a huge effort to investigate whether the ETH can be invoked to explain thermalisation in concrete Hamiltonian models^43^,^44^,^45^,^46^,^47^,^48^,^49^,^50^,^51^,^52^,^53^,^54^,^55^,^56^,^57^ and it’s use it is nowadays ubiquitous. For these reasons, we think it is important to aim at putting ETH under a conceptually clear framework. In this sense, the relevance of our work resides in the fact that we obtain the ETH ansatz as a prediction, by using a maximum-entropy principle as starting point. Furthermore, using the proposed notion of thermal equilibrium is already giving concrete benefits. We now have a way of computing observables which satisfy the ETH and this prediction can be tested both numerically and experimentally. Our investigation proves that maximisation of 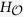 is able to grasp the main intuition behind “thermalisation according to ETH” and the results suggest that it can be the physical principle behind the appearance of ETH.

Further investigation in this direction is certainly needed, but we would to suggest a way in which this new tool can be used to address the long-standing issue of the thermalisation times. From our investigation one can infer that Shannon entropy is a good figure of merit to study the dynamical onset of thermalisation in a closed quantum system. Within this picture, the time-scale at which thermalisation should occur for 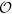 is therefore given by the time-scale at which 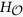 reaches its maximum value. A prediction about the time-scale at which 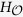 is maximised will translate straightforwardly in a prediction about the thermalisation time.

## Methods

Here we present the full derivation of the equilibrium equations, using the Lagrange multipliers’ technique. Our purpose is to find the distribution which maximises Shannon entropy of an observable 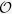. The space on which such an optimisation is formulated is the space of the finite-dimensional density matrices, which is the space of positive semi-definite and self-adjoint matrices. The maximisation is constrained by two equations: the first one accounts for the normalisation of the state while the second one accounts for a fixed value of the average energy.

We present here the derivation, in the general case of a mixed state 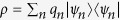 and of a degenerate observable 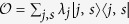, in which 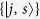 is a complete basis of the Hilbert space. Here are the two constraints:


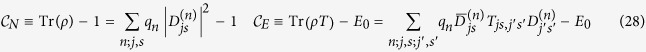


in which 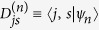. Moreover 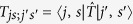. Exploiting Lagrange’s multipliers technique one defines an auxiliary function 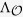, specific for the 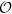 observable, that can be freely optimised


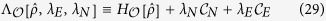


The derivatives with respect to the Lagrange’s multipliers 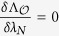 and 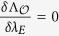 enforce the validity of the constraints 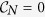 and 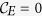, respectively. The derivatives with respect to the overlaps and with respect to the statistical coefficients *q*_*n*_ gives three equations, of which only two are independent: 
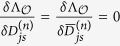
, where


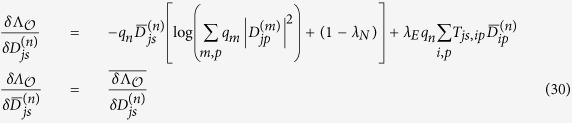


Instead of using these equations, we use the two following independent linear combinations


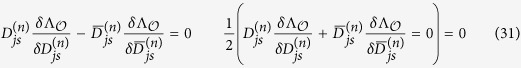


which give the two equilibrium equations that we used in the main text


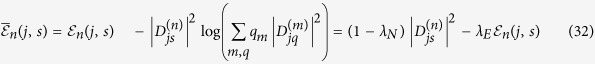


## Additional Information

**How to cite this article:** Anzà, F. and Vedral, V. Information-theoretic equilibrium and observable thermalization. *Sci. Rep.* **7**, 44066; doi: 10.1038/srep44066 (2017).

**Publisher's note:** Springer Nature remains neutral with regard to jurisdictional claims in published maps and institutional affiliations.

## Supplementary Material

Supplementary Information
