# Supplementary material for: Information-theoretic equilibrium and observable thermalization
Source: Sci Rep. 2017 Mar 7;7:44066. doi: 10.1038/srep44066 (PMC5339777; doi:10.1038/srep44066)
Supplement: Supplementary Information [file srep44066-s1.pdf]

"

Information-theoretic equilibrium and observable thermalisation - Supplementary material

F. Anzà[1] and V. Vedral[1, 2, 3, 4]

[1] *Atomic and Laser Physics, Clarendon Laboratory, University of Oxford, Parks Road, Oxford, OX1 3PU, UK*
[2] *Centre for Quantum Technologies, National University of Singapore, 117543 Singapore*
[3] *Department of Physics, National University of Singapore, 2 Science Drive 3, 117551 Singapore*
[4] *Center for Quantum Information, Institute for Interdisciplinary Information Sciences, Tsinghua University, 100084 Beijing, China*

Here we present a study on some properties of the off-diagonal matrix element of an observable which is diagonal in a basis that is unbiased with respect to the Hamiltonian. We will synthetically call such an observable *unbiased observable* and we will show how they satisfy the ETH. We start remembering the ETH ansatz [1] for the matrix element of a generic observable, in the Hamiltonian eigenbasis $|E_\alpha\rangle$:

$$\mathcal{O}_{\alpha\beta}^{\mathrm{ETH}} \approx f_O^{(1)}(\overline{E})\delta_{\alpha\beta} + e^{-\frac{S(\overline{E})}{2}} f_O^{(2)}(\overline{E},\omega)R_{\alpha\beta} \tag{1}$$

where $\overline{E} \equiv \frac{E_\alpha + E_\beta}{2}$, $\omega \equiv E_\alpha - E_\beta$ while $f_O^{(1)}$ and $f_O^{(2)}$ are smooth functions of their arguments. $S(\overline{E})$ is the thermodynamic entropy at energy $\overline{E}$ and $R_{\alpha\beta}$ is a complex random variable with zero mean and unit variance.

Here we used a slightly different notation with respect to the one used in the main text. The basis of the Hilbert space is now called $\{|j\rangle\}$. This is the most natural choice if one wants to investigate such kind of property. On the contrary, the choice of explicitly taking into account the degeneracies of eigenvalues of an observable in such basis $\{|j,s\rangle\}$ is the most natural choice, if one wants to investigate properties of observables rather than bases. We remember the condition of two mutually unbiased basis $\langle j|E_\alpha\rangle = \frac{e^{i\theta_{j\alpha}}}{\sqrt{D}}$. Moreover, the equation to study the off-diagonal matrix elements of an unbiased observable is

$$\mathcal{O}_{\alpha,\beta} = \frac{1}{\mathcal{D}} \sum_j \lambda_j e^{i(\theta_{j\beta} - \theta_{j\alpha})} = \frac{1}{\mathcal{D}} \sum_j \lambda_j \cos(\omega_j^{\alpha\beta}) + \frac{i}{\mathcal{D}} \sum_j \lambda_j \sin(\omega_j^{\alpha\beta}) \tag{2}$$

Where $\mathcal{D}$ is the Hilbert space dimension while λ_j are the eigenvalues of $\mathcal{O}$. In the right-hand side we defined the following short-hand notation for the phase differences: $\omega_j^{\alpha\beta} = \theta_{j\beta} - \theta_{j\alpha}$. We would also like to point out that even though $\{|j\rangle\}$ is a complete basis, the values of λ_j can be highly degenerate in the quantum number j. This is indeed what we expect from a local observable, for example $\sigma_1^{(z)} \otimes \mathbb{I}_{N-1}$, in which $\mathbb{I}_{N-1}$ is the identity matrix over all the qubits but the first one. In this case a basis of the Hilbert space is given by the tensor product between the eigenbasis of $\sigma_1^{(z)}$ and generic basis on rest of the system: $\{|a_j\rangle\} = \{|\pm\rangle \otimes |b_k\rangle\}$. Therefore the eigenvalues will be highly degenerate in such a basis:

$$\sigma_1^{(z)} \otimes \mathbb{I}_{N-1} = \sum_j \lambda_j |a_j\rangle\langle a_j| = \sum_k |+\rangle\langle +| \otimes |b_k\rangle\langle b_k| - |-\rangle\langle -| \otimes |b_k\rangle\langle b_k| \tag{3}$$

Indeed if we choose a specific ordering for the vectors $\{|\pm\rangle|b_k\rangle\}$ we will have

$$|a_j\rangle = \begin{cases} |+\rangle|b_j\rangle & \forall\, j = 1,\ldots,\frac{\mathcal{D}}{2} \\ |-\rangle\left|b_{j \mathrm{mod} \frac{\mathcal{D}}{2}}\right\rangle & \forall\, j = \frac{\mathcal{D}}{2}+1,\ldots,\mathcal{D} \end{cases} \qquad \Longrightarrow \qquad \lambda_j = \begin{cases} +1 & \forall\, j = 1,\ldots,\frac{\mathcal{D}}{2} \\ -1 & \forall\, j = \frac{\mathcal{D}}{2}+1,\ldots,\mathcal{D} \end{cases}$$

The prototypical quantity of which we are studying the general properties can be written as $\sum_j \lambda_j e^{i\omega_j^{\alpha\beta}}$. We are interested in proving that the off-diagonal matrix elements are much smaller that the diagonal ones $\mathcal{O}_{\alpha\beta} \ll \mathcal{O}_{\alpha\alpha}$, therefore we are looking for the mechanisms that can lead to the following estimate:

$$\begin{matrix} \sum_j \lambda_j \cos \omega_j^{\alpha\beta} \\ \sum_j \lambda_j \sin \omega_j^{\alpha\beta} \end{matrix} \ll \sum_j \lambda_j$$

There are several ways in which this can happen and they all represents different physical situations in which a thermal observable will satisfy the ETH. We are now going to mention some of the them:

- *Stationary Phases* - If λ_j is a sufficiently regular function and the $e^{i\omega_j^{\alpha\beta}}$ are quickly oscillating, the value of the sum will be drastically reduced by the rapid oscillations
- *Uncorrelated sequences* - If $\{\lambda_j\}$ and $\left\{e^{i\omega_j^{\alpha\beta}}\right\}$ are uncorrelated $\sum_j \lambda_j e^{i\omega_j^{\alpha\beta}} \approx \left(\sum_j \lambda_j\right)\left(\sum_j e^{i\omega_j^{\alpha\beta}}\right)$. Therefore if one can show that the absolute value of second term is small with respect to one, the result will follow
- *Central limit theorem* - If λ_j is a sufficiently regular function and one can prove that the $e^{i\omega_j^{\alpha\beta}}$ are randomly distributed, it is possible to invoke the central limit theorem and the desired result will follow.

For discussions on the statistical properties of the off-diagonal matrix element of some observables, within some specific Hamiltonian models we suggest the following references [2–6]. We are particularly interested in the third possibility since it has been suggested that the $e^{-\frac{S(\overline{E})}{2}} \sim \frac{1}{\sqrt{\mathcal{D}}}$ scaling behaviour of the off-diagonal matrix elements in Eq.(1) can be a signature of the central limit theorem. More concretely, if we call $|j\rangle$ the eigenstates of an observable $\hat{\mathcal{O}}$, $|E_\alpha\rangle$ the eigenstates of the Hamiltonian and $c_j^{(\alpha)} \equiv \langle j|E_\alpha\rangle$ we have

$$\mathcal{O}_{\alpha\beta} = \sum_j \mathcal{O}_j c_j^{(\alpha)} c_j^{*(\beta)} \tag{4}$$

In [5, 6] the $\frac{1}{\sqrt{\mathcal{D}}}$ scaling behaviour was numerically observed for the off-diagonal matrix elements of some local observables. This led the authors to conjecture that the coefficients $c_j^{(\alpha)}$ are (pseudo-)randomly distributed and some heuristic arguments were used to ascribe the observed scaling behaviour to the validity of the central limit theorem in Eq. (4). For a local observable, the eigenvalues $\mathcal{O}_j^{(loc)}$ are highly degenerate, therefore the sum in Eq. (4) can be split into a small (sub-extensive) number of different sums. If the coefficients $c_j^{(\alpha)}$ behave as independent and randomly distributed, to each one of the different sums it is possible to apply the central limit theorem and to conclude that their value is well described by a gaussian distribution with zero mean and $\frac{1}{\sqrt{\mathcal{D}}}$ variance. We are now going to show that such an hypothesis is true for all the basis which are unbiased with respect to the Hamiltonian basis.

Let's now turn our attention to the study of the distribution of the phases $\theta_{j\alpha}$. All the computations have been performed using Mathematica 10. We first need an algorithm to compute all the mutually unbiased basis in a fixed dimension Hilbert space[7–9]. Two main algorithms which are commonly used are based on the so-called Weyl-group method and the choice between them depends on whether the dimension of the Hilbert space is a prime number p or a power of a prime number p^r. Since the algorithm which can be used in the first case is much simpler, we will study Hilbert spaces with growing dimensions, but always in the case of prime numbers. We observe that for one of the most important cases (qubit systems, $\mathcal{D} = 2^N$) we have an algorithm to explicitly construct such a set of basis. Moreover, from the physical point of view we think that this choice does not limit the validity of our results since it is highly unlikely to observe much differences in thermalization among Hilbert spaces with prime numbers dimensions, power of a prime numbers and generic dimensions. There is no algorithm to compute this set of basis for a Hilbert space of generic dimension.

The Weyl-group method in the case of a prime number dimension [7] can be easily implemented in Mathematica. Starting from the Hamiltonian eigenbasis $\{|E_\alpha\rangle\}$, we construct the following unitary operators:

$$\hat{X} = \sum_{\alpha=0}^{\mathcal{D}-1} |E_{\alpha+1}\rangle \langle E_\alpha| \qquad \hat{Z} = \sum_{\alpha=0}^{\mathcal{D}-1} \omega^\alpha |E_\alpha\rangle \langle E_\alpha| \qquad \omega = e^{i\frac{2\pi}{\mathcal{D}}} \tag{5}$$

It has been proven that the eigenbasis of the following $\mathcal{D}+1$ unitary operators are mutually unbiased[7–9]:

$$\left\{\hat{M}^k\right\}_{k=0}^{\mathcal{D}} : \left\{\hat{Z}, \hat{X}, \hat{X}\hat{Z}, \hat{X}\hat{Z}^2, \ldots, \hat{X}\hat{Z}^{\mathcal{D}-1}\right\} \tag{6}$$

Using a canonical representation for the Hamiltonian vector basis $|E_\alpha\rangle = (0, \ldots, 1_\alpha, \ldots, 0)$, we explicitly computed the matrix elements of this set of operators, diagonalised them and found their eigenbasis $\mathcal{B}_k$, written in term of the Hamiltonian eigenbasis. This allowed us to numerically investigate the distribution of the phases $\theta_{j\alpha}^{(k)}$ which arise from the basis $\mathcal{B}_k$

$$\mathcal{B}_k = \left\{\left|m_j^{(k)}\right\rangle\right\}_{j=1}^{\mathcal{D}} \qquad \left|m_j^{(k)}\right\rangle = \sum_{\alpha=1}^{\mathcal{D}} F_\alpha^{(j,k)} |E_\alpha\rangle \qquad \left\langle m_j^{(k)} | E_\alpha \right\rangle = F_\alpha^{(j,k)} = \frac{e^{i\theta_{j\alpha}^{(k)}}}{\sqrt{\mathcal{D}}} \tag{7}$$

and therefore to study the statistical behaviour of the following quantities:

$$C_j^{\alpha\beta} \equiv \cos\left(\omega_j^{\alpha\beta}\right) \qquad S_j^{\alpha\beta} \equiv \sin\left(\omega_j^{\alpha\beta}\right) \tag{8}$$

When the dimension of the Hilbert space is sufficiently high, there are no qualitative differences among the distributions of the phases in different basis, therefore we will only show the graphs for a given basis $\mathcal{B}_1$. The randomness of the phases can be seen already for relatively small dimensions such as $\mathcal{D} = 149$. We will present the results for $\mathcal{D} = 941$. Fig. (1) is representative for the behaviour of the phases $\theta_{j,\alpha}^{(k)}$

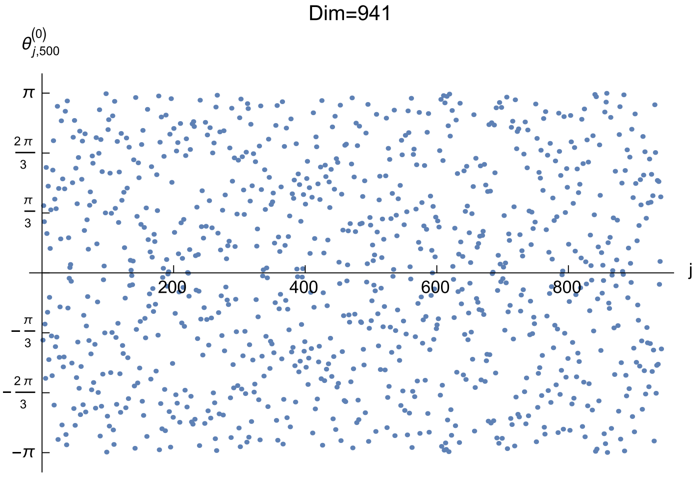

Figure 1: Here we show the behaviour of the phases $\theta_{j,500}^{(1)}$ for an dimension 941 Hilbert space. No qualitative differences can be observed varying the basis $k = 0, 1, \ldots, 940$ or varying the eigenstate under scrutiny $\alpha = 1, 2, \ldots, 500, \ldots, 941$. Small differences can be observed when the eigenstate is at the edge of the spectrum $\alpha = 1, 2, 940, 941$, however the final conclusion on the fact that $\theta_{j,\alpha}^{(k)}$ is not correlated to j holds.

In order to evaluate how the $\theta_{j,\alpha}^{(k)}$ are distributed, we first prove that there is no correlation between them and their labeling j and afterwards study their distribution. We use two statistical tests (Pearson and Spearman[13]) of correlation between the sequences $\left\{\theta_{j,\alpha_0}^{(k_0)}\right\}$ (α_0 and k_0 fixed) and $\{j\}$. The final conclusions of the tests are the same, regardless the basis under study (index k_0) or the Hamiltonian eigenstate (index α_0):

The hypothesis that the correlation coefficient is more than 0.1 is rejected at 5% significance level.

We conclude that for every k_0 and α_0, the sequences $\left\{\theta_{j,\alpha_0}^{(k_0)}\right\}_j$ and $\{j\}_j$ are not correlated or only weakly correlated. We now study the distribution of $S_j^{\alpha\beta}$ and $C_j^{\alpha\beta}$ in order to understand their behaviour and test them

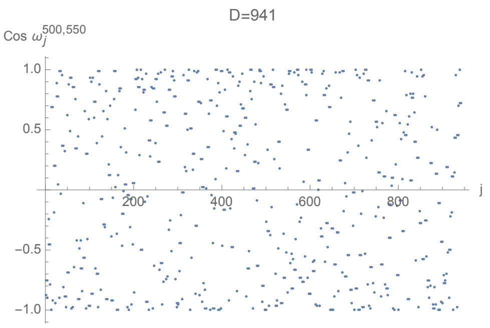

Figure 2: Here we show the behaviour of $\sin\omega_j^{500,550}$, for the basis $k=1$ and for a dimension 941 Hilbert space. No qualitative differences can be observed varying the basis $k=1,\dots,941$. Small differences can be observed when the difference $\alpha-\beta$ is small, however the final conclusion on the fact that $S_j^{\alpha\beta}$ are randomly distributed still holds.

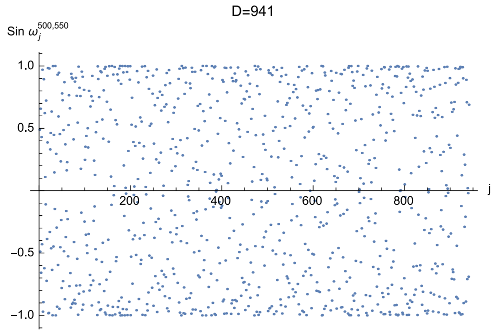

Figure 3: Here we show the behaviour of $\cos\omega_j^{500,550}$, for the basis $k=1$ and for a dimension 941 Hilbert space. No qualitative differences can be observed varying the basis $k=1,\dots,940$. Small differences can be observed when the difference $\alpha-\beta$ is small, however the final conclusion on the fact that $C_j^{\alpha\beta}$ are randomly distributed still holds.

against the hypothesis that they are derived from a constant distribution for the $\omega_j^{\alpha\beta}$. In order to do so we used a Monte Carlo method. We compared the distributions for $S_j^{\alpha\beta}$ and $C_j^{\alpha\beta}$ that we have against a sample produced with the assumption that $\theta_{j,\alpha}^{(k)}$ are identically distributed random variables, with probability distribution $p(\theta)=\frac{1}{2\pi}, \quad \forall\,\theta\in[-\pi,\pi]$. The comparison has been performed using the Chi-Square test [13], with a 5% significance level.

We fixed the basis under study ($k=k_0$), performed the χ^2 test for each pair of indices $\alpha_0,\beta_0=1,\dots,941$. Small differences can be observed, for different pairs and with different basis, however the conclusion is always the same, for every k_0 and each pair α_0 and β_0:

The hypothesis that we can treat $\mathbf{S_j^{\alpha_0\beta_0}},\mathbf{C_j^{\alpha_0\beta_0}}$ as independent and randomly distributed variables is accepted at 5% significance level.

We provide the numeric value of the averages of the Chi-Squares $\overline{\chi}_S,\overline{\chi}_C$ and variances σ_S^2,σ_C^2 of the Chi-Square, over different Hamiltonian eigenstates, respectively for $S_j^{\alpha\beta}$ and for $C_j^{\alpha\beta}$

$$\overline{\chi}_S\equiv\frac{1}{D(D-1)}\sum_{\alpha\neq\beta}\chi_{\alpha\beta}^S=0.96596 \qquad \sigma_S^2\equiv\frac{1}{D(D-1)}\sum_{\alpha\neq\beta}\left(\chi_{\alpha\beta}^S-\overline{\chi}_S\right)^2=0.0039 \tag{9}$$

$$\overline{\chi}_C\equiv\frac{1}{D(D-1)}\sum_{\alpha\neq\beta}\chi_{\alpha\beta}^C=0.96445 \qquad \sigma_C^2\equiv\frac{1}{D(D-1)}\sum_{\alpha\neq\beta}\left(\chi_{\alpha\beta}^S-\overline{\chi}_S\right)^2=0.0041 \tag{10}$$

CONCLUSIONS

We conclude that it is possible to treat $\sin\left(\theta_{j,\alpha}^{(k)}-\theta_{j,\beta}^{(k)}\right)$ and $\cos\left(\theta_{j,\alpha}^{(k)}-\theta_{j,\beta}^{(k)}\right)$ as independent and identically distributed random variables, for each $(\alpha,\beta)\ :\alpha,\beta=1,\dots,\mathcal{D}$ but $\alpha\neq\beta$ and for every $k=1,\dots,\mathcal{D}$.

---

[1] M. Srednicki, *The approach to thermal equilibrium in quantized chaotic systems*, J. Phys. A **32** 1163 (1999)
[2] R. Steinigeweg, J. Herbrych, and P. Prelovsek, *Eigenstate thermalization within isolated spin-chain systems*, Phys. Rev. E **87**, 012118 (2013).

[3] E. Khatami, G. Pupillo, M. Srednicki, M. Rigol, *Fluctuation-Dissipation Theorem in an Isolated System of Quantum Dipolar Bosons after a Quench*, Phys. Rev. Lett. **111** 050403 (2013)

[4] N. P. Konstantinidis, *Thermalization away from integrability and the role of operator off-diagonal elements*, Phys. Rev. E **91**, 052111 (2015)

[5] W. Beugeling, R. Moessner, M. Haque, *Finite-size scaling of eigenstate thermalization*, Phys. Rev. E **89**, 042112 (2014)

[6] W. Beugeling, R. Moessner, M. Haque, *Off-diagonal matrix elements of local operators in many-body quantum systems*, Phys. Rev. E **91**, 012144 (2015)

[7] I. Bengtsson, *Three way to look at Mutually Unbiased Basis*, AIP Conf. Proc. 889, 40 (2007)

[8] S. Bandyopadhyay, P. O. Boykin, V. Roychowdhury, F. Vatan, *A new proof for the existence of mutually unbiased bases*, ArXiv:quantum-ph/0103162v3

[9] J. Lawrence, C. Brukner, A. Zeilinger, *Mutually unbiased binary observable sets on N qubits*, Phys. Rev. A 65, 032320

[10] Kraus - *Complementary observables and uncertainty relations*, Phys. Rev. D **35** 3070 (1987)

[11] Wehner, Winter - *Entropic uncertainty relations - a survey*, New J. Phys. **12** (2010) 025009

[12] Massen, Uffink - *Generalized Entropic Uncertainty Relations*, Phys. Rev. Lett. **60** 1103 (1988)

[13] J. K. Taylor, C. Cihon, *Statistical Techniques for Data Analysis*, Second Edition, Chapman & Hall (2004)
